# Diagnostic Value of Preoperative Needle Biopsy for Tumor Grading Assessment in Hepatocellular Carcinoma

**DOI:** 10.1371/journal.pone.0144216

**Published:** 2015-12-14

**Authors:** Lijun Wang, Jianguo Wang, Xuanyu Zhang, Jie Li, Xuyong Wei, Jun Cheng, Qi Ling, Haiyang Xie, Lin Zhou, Xiao Xu, Shusen Zheng

**Affiliations:** 1 Department of Pathology, First Affiliated Hospital, Zhejiang University School of Medicine, Hangzhou, Zhejiang, China; 2 Division of Hepatobiliary and Pancreatic Surgery, Department of Surgery, First Affiliated Hospital, Zhejiang University School of Medicine, Hangzhou, Zhejiang, China; 3 Key Lab of Combined Multi-Organ Transplantation, Ministry of Public Health, Hangzhou, Zhejiang, China; 4 Collaborative innovation center for diagnosis and treatment of infectious diseases, Zhejiang University, Hangzhou, Zhejiang, China; University of Modena & Reggio Emilia, ITALY

## Abstract

**Background:**

Needle core biopsy (NCB) is one of the most widely used and accepted methods for the diagnosis of focal hepatic lesions. Although many studies have assessed the diagnostic accuracy of NCB in predicting the tumor grade, it is still under debate.

**Objective:**

To identify the influence of number of biopsies on NCB diagnostic accuracy.

**Methods:**

153 patients with HCC were selected from patients who received preoperative NCB under the guidance of ultrasonography in our hospital. The diagnostic reference standard was the surgical pathologic diagnosis.

**Results:**

Using a 3-tier grading scheme (well, moderate and poor), the accuracy of NCB has no significant differences among different number of passes in HCC ≤5cm. For HCC >5≤8cm, the increasing number of passes could increase the diagnostic accuracy (63.3%, 81.8%, and 84.8% for passes one, two, and three, respectively). While in HCC>8cm, the diagnostic accuracy of passes one, two, and three were 62.1%, 69%, and 75.8%, respectively.

**Conclusions:**

The accuracy of NCB in assessing tumor grading associated with tumor size and number of passes. Meanwhile, a minimum of two passes should be performed to get better accuracy in patients with HCC >5cm.

## Introduction

Hepatocellular carcinoma (HCC) is one of the leading causes of cancer-associated mortality globally [1, 2]. The incidence of HCC in the developing countries remains high and the incidence in the developed countries is progressively increasing. At present, the screening of HCC in at-risk patients relies on measuring the serum level of alpha-fetoprotein (AFP) and ultrasound examination [3]. However, these screening methodologies are unable to identify the molecular typing of HCC and preoperative tumor grade; AFP’s sensitivity in screening of HCC is very limited as increased level of AFP has been reported in many other liver diseases as well [4, 5]. Tumor grade was could be a risk factor for relapse of tumor and it could impact the survival of patients after resection of HCC [6]. Hence, correct diagnosis of tumor grade is important for appropriate selection of therapeutic strategy. Currently, liver transplantation (LT) is the best therapeutic strategy for patients with HCC. However, the Milan criteria [7] select candidate for LT without any consideration of the tumour histology, only based on strict morphologic evaluations. Indeed, the histological examination could play a critical role in the assessment of the biological behavior of the tumor and in determining the patient outcome after LT[8]. According to Hangzhou criteria, determining the degree of differentiation of tumor is one of the important screening criterion to choose the suitable candidates for LT [9]. Accurate tissue diagnosis is usually achieved via percutaneous sampling of tumors. Needle core biopsy (NCB) is a widely used and accepted methods for obtaining tumor tissue samples.

Large core tissue samples could be obtained easily from various parts of a nodule and affected organs through NCB for histological and morphological examinations, which could play an important role in subtyping HCC and assessing the biological behavior of the tumor. Moreover, core biopsy samples can be used in special diagnostic procedures such as immunohistochemistry, biomolecular, and receptor analyses[10], which further helps to predict the early and late stage recurrence of tumor and the overall survival[11]. NCB is a widely used method to obtain the pre-operative tissue specimen for both HCC diagnosis and grading.

Only few studies assessed the concordance of preoperative diagnosis using NCB with postoperative pathology reports of tumors; however, as the results were inadequate and diversified, no true consensus could be arrived [12]. The present study aimed to assess the accuracy of percutaneous NCB in tumor grading of patients who underwent liver resection for a single nodule HCC and to identify whether the more number of NCB passes could increase the diagnostic accuracy.

## Materials and Methods

### Patients

A total of 153 patients who were preoperatively diagnosed with HCC through NCB from 2010 to 2012 in the First Affiliated Hospital of Zhejiang University (Hangzhou, China) were retrospectively evaluated. The patients underwent NCB before liver resection for HCC. The study inclusion criteria were as follows: (1) patients with a single nodule HCC, (2) availability of both histological biopsy data and pathology report of surgical specimens for each patient, (3) tumor near the surface. The study exclusion criteria were as follows: (1) Patients received loco-regional therapies, (2) patients with multiple nodules, or (3) insufficient sampling specimens for immunostaining. Routine coagulation tests were performed before the NCB to assess the suitability and associated patient’s safety. All the patients were well informed about the NCB procedure. The written informed consent was obtained from all the participated patients. Standard clinicopathologic data including age, sex, AFP level, tumor size, vascular invasion (major and microscopic), presence of cirrhosis, and histologic grade were collected. The present study was approved by the Institutional Review Board of the First Affiliated Hospital of Zhejiang University.

### Biopsy procedure and samples management

Preoperative NCB was performed using a semiautomatic 19-gauge Trucut core biopsy needle (TSK Laboratory, Japan). The NCBs were performed by an interventional radiologist using an ultra sound-guided technique. Before the procedures, routine coagulation tests were collected and evaluated. Each patients was individually assessed to determine the best access route according to imaging examination. Biopsies were performed with a standard sterile technique and with the patient under local anesthesia with 2% lidocaine. For a patient with a liver nodule, three biopsies were carried out within the lesionand the obtained biopsy specimens were fixed in 10% buffered formalin. Three passes were performed in the middle and both sides of each nodule, respectively. Additionally, three biopsies were carried out during a same operation and the entire process was completed in several minutes. Computed tomography scans were performed immediately after all the procedures to verify the bleeding or accidental injuries. Patients remained under observation for ≥ 1 h after the procedure. After fixation, the biopsy specimens were are labeled and embedded in paraffin separately.

### Histological diagnosis

At our institution, biopsy and surgical specimens are evaluated by two independent pathologists specializing in liver disease. When the two pathologists got two different conclusions, they need to discuss each other to get a final results. Surgically resected specimens were used as the gold standard for diagnosis of tumor grading. The degree of pathological differentiation of HCC was identified using Edmonson-Steiner classification[13]: G1–G2: well differentiated; G3: moderately differentiated; and G4: poorly differentiated. When observed the same differentiation grade in the entire lesion, HCC was regard as a homogeneously-differentiated carcinoma. Likewise, HCC was considered as a heterogeneously-differentiated carcinoma if several differentiation grades were observed in the lesion.

### Immunohistochemistry

To specifically study the correlation between biomarkers and HCC histological grading, paraffin embedded biopsy sample sections were stained with antibody against Ki67. The detailed procedure were described as previously reported.[14] Hematoxylin and Eosin (H&E) staining was performed according to standard protocol.

### Statistical analysis

The concordance of tumor grade assessment between the pre-operative NCB and the postoperative pathological staging was evaluated using the k-statistics [15]. Differences in diagnostic accuracy (1 pass vs. 2 passes vs. 3 passes) were evaluated by u-test and Fisher's exact test when appropriate. A p <0.05 were considered statistically significant. The SPSS 13.0 software was used to statistically analyses.

## Results

### Clinicopathologic characteristics and complications

The clinicopathologic characteristics of the 153 patients are summarized in Table 1. There were 126 men and 27 women. The mean patient age was 52.37±11.4 years (range: 26–89 years) and the mean tumor size was 5.57±3 cm (range 2–15 cm). A total of 111 patients (72.5%) had cirrhosis. Of the 153 patients 124 (81%) had viral hepatitis B, 5 (3.3%) patients had hepatitis C, and the remaining 24 patients (15.7%) were cryptogenic. A total of 39 patients (25.5%) had the AFP levels of >400 ng/ml. No complications of NCB were recorded in this study.

**Table 1:** Clinical and Pathologic Features of Patients (n = 153).

Variable	Value
Mean age (years)	52.37±11.4
Sex n (%)	
Male	126(82.4%)
Female	27(17.6%)
Mean tumor size / Larger tumor (cm)	5.57±3/15
Tumor size n (%)	
≤3cm	37(24.2%)
>3cm≤5cm	54(35.3%)
>5cm≤8cm	33(21.6%)
>8cm	29(18.9%)
Hepatic cirrhosis n (%)	
No	42(27.5%)
Yes	111(72.5%)
Aetiology n (%)	
Hepatitis B	124(81%)
Hepatitis C	5(3.3%)
Cryptogenic	24(15.7%)
Microvascular Invasion	59
α fetoprotein levels n (%)	
1–20	73(47.7%)
20–400	41(26.8%)
>400	39(25.5%)

### Histologic data

**Fig 1:**
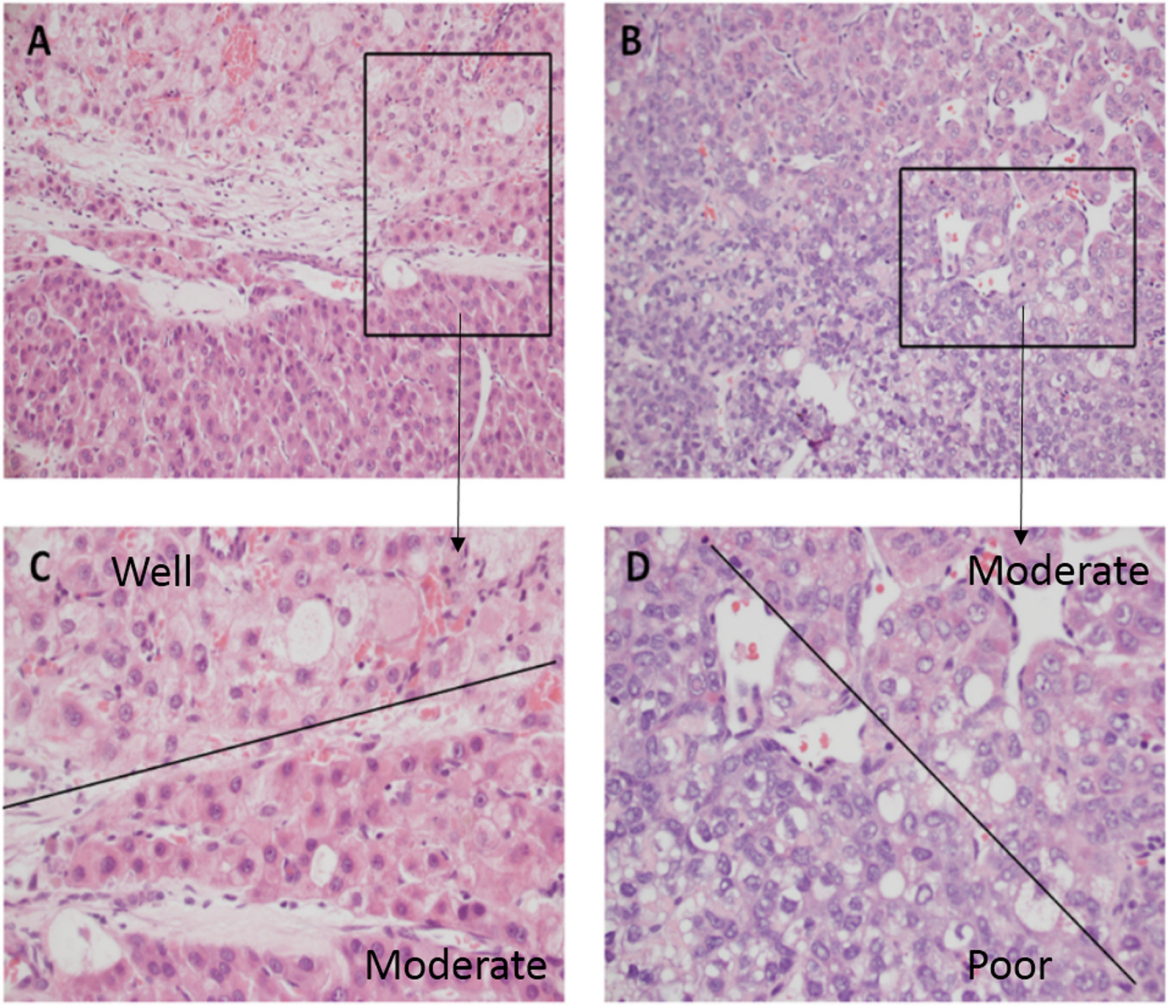
Tumor grade in HCC are commonly very heterogeneous. C, A portion of tumor is grade 1(the morphology of tumor cells were close to the normal liver cells, with augmentation of nucleus/cytoplasm) while an adjacent area is grade 2 (which contains markedly enlarged oncocytic hepatocytes with some nuclear pleomorphism and angulation). D, An area of grade 2 is adjacent to an area of grade 3 (the cell shape are quite irregular, tumor cells show marked pleomorphism). C and D demonstrated the higher magnification from the area of the box in A and B, respectively. (A–B: 200 ×magnification; C-D: with 400 × magnification).

Heterogeneous HCCs are more common than the homogeneously-differentiated HCCs. As shown in Fig 1, several differentiation grades were simultaneously present in the overall lesion. Therefore, the judgment of histological grading between different pathologists may discrepant. In the present study, a total of 19 cases were discrepant. For these discrepant cases, the two pathologists discussed each other and made a final conclusion by setting up a threshold. In this study, 10% was considered as the boundary value, when the proportion of a higher grade was greater than or equal to 10% in heterogeneous tumors, the higher possible grade was considered as the final diagnostic results. Postoperative pathological examination revealed that 69 out of 153(45.1%) HCCs presented intratumoral heterogeneity. On relating the nodule size with tumor heterogeneity, 11 out of 37 HCCs (29.7%) with nodule size ≤3cm had heterogeneity, whereas in the other sub-categories of HCCs with nodule sizes of >3 and ≤5cm, >5 and ≤8cm, and >8cm, the percentage of intratumoral heterogeneity were 42.6%, 54.5%, and 58.6%, respectively. The results suggested that the risk of histologic heterogeneity increases with increasing nodular size of HCCs. The rate of heterogeneity existed significant difference between HCCs ≤3 cm and HCCs>8cm (p = 0.0217).

The presence of microvascular invasion in the surgical specimens was observed in 59 HCCs (59/153, 38.6%). According to the tumor grade, the percentage of microvascular invasion HCC in the surgical specimens was as follows: 7 out of 48 (14.6%) well differentiated HCCs, 45 out of 91 (49.5%) moderately differentiated HCCs, and 7 out of 14 (50%) poorly differentiated HCCs.

On comparing the AFP level with tumor differentiation, 60.4% patients with well-differentiated HCC had AFP ≤20ng/ml. The percentage is much higher than moderately and poorly differentiated HCCs (39.5% and 35.7%, respectively).

### Concordance of tumor grade assessment

**Fig 2:**
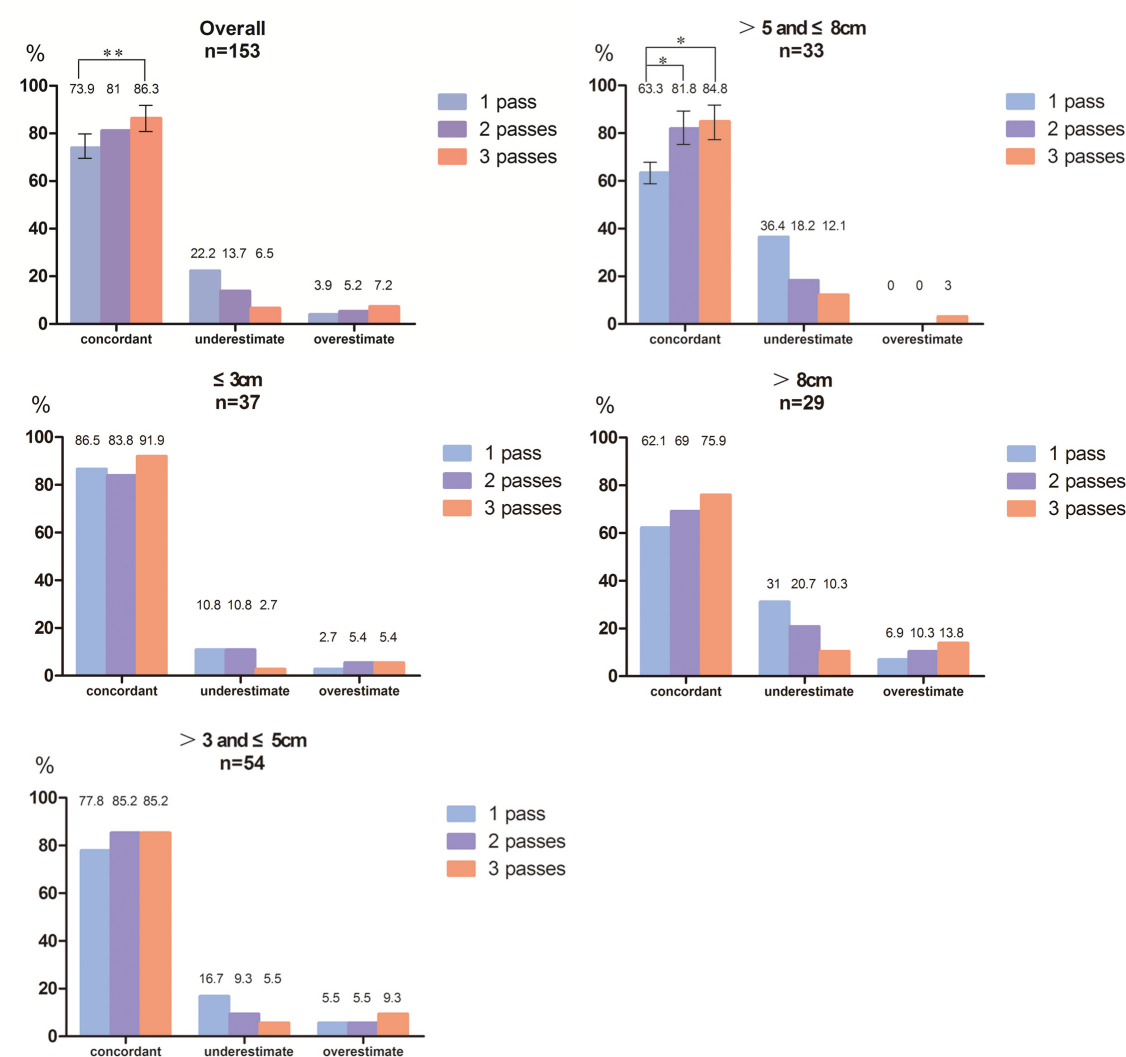
Concordance of tumor grade assessment between needle core biopsy (NCB) and surgical specimen. The figure indicates the diagnostic accuracy of tumor grade between NCB and surgical specimen in the overall population and according to tumor size. The accuracy was gradually increasing along with the increase of the number of NCB passes. *, p<0.05 and **, p<0.01.

The concordance between preoperative NCB and final pathologic tumor grade using a 3-tier grading scheme are presented in Table 2. The diagnostic accuracy of 1 pass, 2 passes, and 3 passes were 73.9%, 81%, and 86.3%, respectively. The result suggested that the overall accuracy was gradually increasing along with the increase of the number of NCB passes. In addition, performing preoperative NCB 3 passes could significantly elevate accuracy compared with 1 pass (p = 0.0033). Furthermore, patients were assigned into different sub-categories according to HCC size. Fig 2 indicates the degree of concordance between preoperative NCB and final pathologic tumor grade in the different sub-categories.

**Table 2:** Concordance of Tumor Grade on Preoperative Needle Core Biopsy versus Final Surgical Pathology Using 3-Tier Grading System.

NCB	Surgical specimen						
1 pass	Well	Moderate	Poor	Total	K statistic	P.value	accuracy
Well	45	22	0	64	0.52	0.000	73.9%
Moderate	3	66	12	85			
Poor	0	3	2	4			
Total	48	91	14	153			
2 passes							
Well	43	12	0	53	0.644	0.000	81%
Moderate	5	76	9	95			
Poor	0	3	5	5			
Total	48	91	14	153			
3 passes							
Well	41	6	0	47	0.745	0.000	86.3%
Moderate	7	81	4	92			
Poor	0	4	10	14			
Total	48	91	14	153			

For HCC with ≤3cm or >3 and ≤5cm, no significant difference was observed among the diagnostic accuracy of NCB with passes one, two, and three, respectively. For HCC >5 and ≤8cm, the accuracy of one pass was 63.3% and the accuracy of passes two and three were 81.8% and 84.8% (P = 0.0495 and 0.0244), respectively. While in the group of HCC >8cm, the diagnostic accuracy of passes one, two, and three were 62.1%, 69%, and 75.8% (P>0.05), respectively. Hence, it was concluded that the concordance of tumor grade between the NCBs and the surgical specimens were higher in early-stage (<3cm) HCC, and for HCC ≤5cm, one pass NCB was enough to get a high accuracy. However, NCB must be performed at least twice to get better accuracy than only one pass in the group of HCC >5 and ≤8cm. Meanwhile, increasing the number of NCB passes could improve the NCB diagnostic accuracy for HCC >8cm.

For heterogeneous tumors (69/153 patients), it was found that the increasing number of NCB passes could significantly improve the NCB diagnostic accuracy. The diagnostic accuracy of passes one, two, and three were 27.5%, 53.6%, and 76.8% (P<0.001), respectively.

The long-term survival is similar between patients with well and moderately-differentiated tumors after LT, whereas the patients with poorly-differentiated tumors could have a significantly worse outcome following LT. Hence, a two-tier grading scheme (well- and moderately-differentiated vs. poorly differentiated) was established and the results of diagnostic accuracy were shown in Table 3. Using this two-tier grading scheme, a good diagnostic accuracy could be achieved according to HCC size in the different sub-categories, and there was no obvious difference among different number of passes.

**Table 3:** Accuracy of Tumor Grade on Preoperative Needle Core Biopsy Versus Final Surgical Pathology Using 2-Tier Grading System (well-/moderately differentiated vs. poorly differentiated).

		Accuracy (%)		
	number	1 pass	2 passes	3 passes
Total	153	90.1	92.1	94.8
≤3 cm	37	91.9	91.9	97.3
>3 cm and ≤5 cm	54	96.3	96.3	98.1
>5cm and ≤8 cm	33	84.8	90.9	90.9
>8 cm	29	82.7	86.2	82.7

### The diagnostic accuracy of NCB in homogeneously differentiated and heterogeneous HCCs

In order to investigate the influence of intratumor heterogeneity on diagnostic accuracy of NCB, the diagnostic accuracy of NCB between homogeneously differentiated and heterogeneous HCCs were evaluated. In homogeneously-differentiated HCCs, there was no significant difference among the different number of passes (83.3%, 88.1% and 85.7%, respectively, for passes one, two, and three). However, for HCC with intratumor heterogeneity, the diagnostic accuracy of NCB was raised significantly with increasing number of passes. The accuracy of passes one, two, and three in the heterogeneous HCCs were 55.1%, 72.5%, and 87% (P<0.001), respectively.

### Expression of Ki67 in HCC with different histologic grade

Ki-67 is an important index in diagnosis of tumors, for it can reflect cellular proliferative activity. Meanwhile, the tumors with poorly differentiation usually has a faster growth rate. Hence, we detected the expression of Ki-67 in the biopsy specimens and assessed whether Ki-67 can be used as a possible surrogate biomarker of histologic grade. However, the present study results indicated that there was no correlation between Ki-67 expression and histological grading (S1 Fig). Therefore, Ki-67 may not an ideal marker of HCC histological grade. Additional biomarkers will be investigated in further study.

## Discussion

At present, LT is one of the intervention for patients with HCC [16], and selecting the appropriate patients who can benefit from LT is crucial for better outcome. LT is superior to resection because of lower tumor recurrence rate and long-term survival of patients. However, the selection criteria of LT Candidates have become more strictly over the last decade. In 1996, Mazzaferro V et al established the Milan criteria: only patients with HCC and cirrhosis who had ≤3 tumor nodules with maximum diameter of ≤3 cm or a single tumor ≤5 cm and no clinically apparent signs of vascular invasion were considered acceptable for transplantation. The strict morphologic criteria can improve the survival of patients with HCC post LT to a great extent [7, 17]. However, although some patients beyond the Milan criteria, they may still benefit from transplantation LT [18–21].

Growing experience of LT for HCC has suggested that Milan criteria is too restrictive and fail to include the increasing candidate list, particularly in China [22]. The transplantation selection criteria including preoperative tumor grade for those patients exceeding the Milan criteria have been proposed recently [9, 23, 24]. Therefore, in 2008, Hangzhou criteria was proposed to further help to choose the candidates for LT: (a) total tumor diameter less than or equal to 8 cm; or (b) total tumor diameter more than 8 cm, with histopathologic grade I or II and preoperative AFP level less than or equal to 400 ng/mL. A multicenter study including 6012 patients with HCC undergoing LT from the China Liver Transplant Registry (CLTR) suggested the following: Hangzhou criteria expanded the scope of LT compared to Milan criteria for patients with HCC to 51.5% with significantly better overall and tumor-free survival [25].

The different tumor histologic grade are critical in assessing the prognosis and guiding patient therapy [26, 27]. Currently, NCB is one of the most widely used and accepted methods of needle biopsy. However, as most of the HCCs are heterogeneously-differentiated, the desired diagnostic accuracy cannot be obtained with a single pass NCB of the HCC nodule. NCB is an invasive procedure for the diagnosis of focal liver lesions. Therefore, the present study aimed to identify the balance point between the number of passes and NCB accuracy in HCC.

In the present study, preoperative NCB of HCC was compared with the surgical resection specimen with respect to histologic tumor grading. The results of the present study indicated that the preoperative NCB was significantly related to the number of passes and tumor size. Further, the present study results indicated that NCB could be accurate in assessing the tumor grade of early-stage HCC (≤5cm). For HCC ≤5cm, one pass NCB is enough to get higher accuracy; however, minimum of two passes are to be performed for better accuracy in HCC >5 and ≤8cm.

NCB is the only pre-operative method for obtaining liver biopsies to assess the histological grading of the tumor. However, comparing with the pathology conclusion of the surgical specimen, the accuracy of NCB was still under debate. In Colecchia A et al. study, high overall accuracy (91.4%) and low associated complication rate had been reported for early-stage HCC (<5cm) screening with NCB [6]. Whereas in Pawlik et al. study, it was reported that the preoperative histologic tumor grading through NCB was inaccurate due to its low sensitivity (34%) in assessing the tumor grade in poorly-differentiated HCC [12]; It was concluded that the selection of candidates for LT based on preoperative NCB tumor grade was an inappropriate approach. However, those study findings were just based on a single pass NCB on HCC nodules. Interestingly, in the present study, three NCB passes were performed for each patient in order to assess whether the overall accuracy improved after multiple NCB passes and to evaluate the concordance of NCB versus final surgical specimen. The present study findings suggested that concordance of NCB versus final surgical specimen and the overall accuracy were increased with the increasing number of passes. Using a 3-tier grading scheme for analysis, the overall accuracy was lower than that of Colecchia A et al. even when 3 passes were performed. Meanwhile, the concordance value (0.745 VS 0.72) was higher in comparison with them when 3 passes were performed. Our data suggested that the accuracy of NCB could be elevated by increasing the number of passes. Moreover, the accuracy and concordance of NCB was influenced by the tumor size. The diagnostic accuracy of NCB was higher for smaller HCCs, especially in HCC <3cm. However, the accuracy decreased along with the increase of tumor diameter. In addition, in different subclasses regarding to tumor size, the accuracy obtained from different passes also exist differences. In particularly, in HCC >5 ≤8 cm, 2 passes could significantly increase the accuracy.

Further, in the present study, a 2-tier grading classification was also established: well and moderately differentiated vs. poorly differentiated groups. This grading classification model was chosen because the patients with well- and moderately-differentiated tumors had similar long-term survival after LT. Among the different sub-categories of patients classified based on HCC size, no significant difference in diagnostic accuracy of NCB was observed irrespective of number of passes. Therefore, one pass is adequate to get a better diagnostic accuracy in the screening of patients with HCC for LT. In the present study, the diagnostic accuracy was low at one NCB, and it probably was attributed to the presence of intratumor heterogeneity [15]. In our series, 45.1% of the tumors were heterogeneous. Furthermore, in agreement with others reports [6, 28], our results suggested that the percent of histologic heterogeneity with regard to tumor differentiation increases with increasing diameter of HCCs. For HCC>8cm, the percent of tumors with heterogeneity was as high as 58.6%. Hence, the decreased NCB accuracy in those patients was attributed to greater heterogeneity, even after 3 passes. In addition, it was found that the reduced diagnostic accuracy caused by tumor heterogeneity could be improved with increasing number of passes, especially in HCC >5 ≤8 cm.

Previous studies [29–32] have indicated that microvascular invasion is one of the important predictors of tumor recurrence after LT or hepatic resection. However, microvascular invasion, can only be assessed through histopathological examination of liver specimens. Moreover, tumor histologic grade has also been investigated as a possible index of microscopic invasion. Some studies reported that tumor grade was highly correlated with microvascular invasion. In Esnaola et al study, poorly differentiated tumors were correlated well with microvascular invasion in patients with HCC [33]. Han et al. suggested that the poorly differentiated HCC with mixed histologic grades could determine the prognosis after curative resection of HCC and the microvascular invasion was more frequently observed in poorly differentiated group [34]. In the present study, close relationship was observed between the tumor grade and microvascular invasion. In moderate and poorly differentiated groups, the percentage of microvascular invasion was 49.5% and 50%, respectively, whereas in well-differentiated group, only 7(14.6%) patients presented microvascular invasion. These findings were consistent with previous published data.

In the current study, 3 passes were performed for each patient. Pawliket al. reported that multiple NCBs may be inadvisable because it may associated with an increased incidence of tumor recurrence [12]. Moreover, it was reported that complications of NCB were increased when more than three passes were made [35]. However, no serious NCB complications were observed in the present study. It might be because of the good clinical condition of the patients as well as NCBs were performed by an experienced surgeon. Moreover, increased complications of NCB were reported when it was performed by less-experienced clinicians [36]. The reported rate of dissemination of HCC along the biopsy needle track has ranged between 0% and 5% [37–39]. However, in the present study, no patients with dissemination of the tumor along the needle track was identified. Some researchers have claimed that needle size, number of passes, tumor size, and differentiation grade are some of the risk factors of tumor implantation along the needle track during NCB [39], while others have stated that true risk factors are unidentified. Meanwhile, some investigators have suggested that the spread of the tumor along the needle track can often be treated effectively by local resection and it rarely leads to fatal outcome [40, 41]. In the present study, the patients will be followed up closely to observe the incidence of needle tract seeding following biopsy.

In conclusion, the present study data suggested that the diagnostic accuracy of NCB was associated with tumor size and the number of passes. Using a 3-tier grading scheme, tumor grade of early-stage (<5cm) HCC can be assessed accurately with ultrasound-guided NCB. For HCC ≥5 and <8cm, minimum of two passes are required to obtain a good result in the assessment of tumor grade with NCB. In addition, in the screening of patients with HCC for orthotopic LT, one pass preoperative NCB is adequate to get acceptable diagnostic accuracy of histologic grade of HCC.

## Supporting Information

S1 Fig Representative images of immunohistochemistry of Ki67. A and C: the histological grading of HCC cells were grade 2, the percentage of Ki-67 positive cell were <5% and 10%, respectively. E and G: The histological grading of HCC cells were grade 3 and the percentage of Ki-67 positive cell were 10% and 30%, respectively. I: histological grading of HCC was grade 3 in the overall lesion, whereas Ki-67 positive cell were uneven distributed. B, D, F and H demonstrated the higher magnification of A, C, E and G, respectively. (A, C, E, G and I: 200 ×magnification; B, D, F and H: with 400 × magnification).
(TIF)

## Abbreviations

NCB: Needle core biopsy
HCC: Hepatocellular carcinoma
AFP: a-fetoprotein
LT: Liver transplantation
FNAC: fine needle aspiration cytopathology

## References

[1] Diamond DL, Proll SC, Jacobs JM, Chan EY, Camp DG 2nd, Smith RD, et al HepatoProteomics: applying proteomic technologies to the study of liver function and disease. Hepatology. 2006;44(2):299–308. Epub 2006/07/28. 10.1002/hep.21318 . 16871559

[2] Wu LM, Zhou L, Xu J, Wei BJ, Cheng J, Xu X, et al Lack of association between genetic polymorphisms in cytokine genes and tumor recurrence in patients with hepatocellular carcinoma undergoing transplantation. Hepatobiliary Pancreat Dis Int. 2013;12(1):54–9. Epub 2013/02/09. . 23392799 10.1016/s1499-3872(13)60006-5

[3] Soga T, Sugimoto M, Honma M, Mori M, Igarashi K, Kashikura K, et al Serum metabolomics reveals gamma-glutamyl dipeptides as biomarkers for discrimination among different forms of liver disease. J Hepatol. 2011;55(4):896–905. Epub 2011/02/22. 10.1016/j.jhep.2011.01.031 S0168-8278(11)00159-0 [pii]. . 21334394

[4] Nicholson JK, Lindon JC. Systems biology: Metabonomics. Nature. 2008;455(7216):1054–6. Epub 2008/10/25. 10.1038/4551054a 4551054a [pii]. . 18948945

[5] Arakaki AK, Skolnick J, McDonald JF. Marker metabolites can be therapeutic targets as well. Nature. 2008;456(7221):443 Epub 2008/11/28. 10.1038/456443c 456443c [pii]. . 19037294

[6] Colecchia A, Scaioli E, Montrone L, Vestito A, Di Biase AR, Pieri M, et al Pre-operative liver biopsy in cirrhotic patients with early hepatocellular carcinoma represents a safe and accurate diagnostic tool for tumour grading assessment. Journal of hepatology. 2011;54(2):300–5. 10.1016/j.jhep.2010.06.037 . 21056498

[7] Mazzaferro V, Regalia E, Doci R, Andreola S, Pulvirenti A, Bozzetti F, et al Liver transplantation for the treatment of small hepatocellular carcinomas in patients with cirrhosis. The New England journal of medicine. 1996;334(11):693–9. 10.1056/NEJM199603143341104 . 8594428

[8] Sutcliffe R, Maguire D, Portmann B, Rela M, Heaton N. Selection of patients with hepatocellular carcinoma for liver transplantation. Br J Surg. 2006;93(1):11–8. Epub 2005/12/06. 10.1002/bjs.5198 . 16329080

[9] Zheng SS, Xu X, Wu J, Chen J, Wang WL, Zhang M, et al Liver transplantation for hepatocellular carcinoma: Hangzhou experiences. Transplantation. 2008;85(12):1726–32. 10.1097/TP.0b013e31816b67e4 . 18580463

[10] Thorgeirsson SS, Lee JS, Grisham JW. Molecular prognostication of liver cancer: end of the beginning. J Hepatol. 2006;44(4):798–805. Epub 2006/02/21. doi: S0168-8278(06)00053-5 [pii] 10.1016/j.jhep.2006.01.008 . 16488507

[11] Hoshida Y, Villanueva A, Kobayashi M, Peix J, Chiang DY, Camargo A, et al Gene expression in fixed tissues and outcome in hepatocellular carcinoma. N Engl J Med. 2008;359(19):1995–2004. Epub 2008/10/17. 10.1056/NEJMoa0804525 NEJMoa0804525 [pii]. 18923165 PMC2963075

[12] Pawlik TM, Gleisner AL, Anders RA, Assumpcao L, Maley W, Choti MA. Preoperative assessment of hepatocellular carcinoma tumor grade using needle biopsy: implications for transplant eligibility. Ann Surg. 2007;245(3):435–42. Epub 2007/04/17. 10.1097/01.sla.0000250420.73854.ad 00000658-200703000-00015 [pii]. 17435551 PMC1877015

[13] Shin E, Yu YD, Kim DS, Won NH. Adiponectin receptor expression predicts favorable prognosis in cases of hepatocellular carcinoma. Pathology oncology research: POR. 2014;20(3):667–75. 10.1007/s12253-014-9747-0 . 24619866

[14] Wang J, Xu X, Liu Z, Wei X, Zhuang R, Lu D, et al LEPREL1 Expression in Human Hepatocellular Carcinoma and Its Suppressor Role on Cell Proliferation. Gastroenterology research and practice. 2013;2013:109759 10.1155/2013/109759 24319452 PMC3844253

[15] An FQ, Matsuda M, Fujii H, Tang RF, Amemiya H, Dai YM, et al Tumor heterogeneity in small hepatocellular carcinoma: analysis of tumor cell proliferation, expression and mutation of p53 AND beta-catenin. Int J Cancer. 2001;93(4):468–74. Epub 2001/07/31. 10.1002/ijc.1367 [pii]. . 11477549

[16] Chok K, Chan SC, Fung JY, Cheung TT, Chan AC, Fan ST, et al Survival outcomes of right-lobe living donor liver transplantation for patients with high Model for End-stage Liver Disease scores. Hepatobiliary Pancreat Dis Int. 2013;12(3):256–62. Epub 2013/06/08. . 23742770 10.1016/s1499-3872(13)60042-9

[17] Llovet JM, Bruix J, Fuster J, Castells A, Garcia-Valdecasas JC, Grande L, et al Liver transplantation for small hepatocellular carcinoma: the tumor-node-metastasis classification does not have prognostic power. Hepatology. 1998;27(6):1572–7. Epub 1998/06/10. doi: S0270913998002353 [pii] 10.1002/hep.510270616 . 9620329

[18] Lee Cheah Y, KHC P. Liver Transplantation for Hepatocellular Carcinoma: An Appraisal of Current Controversies. Liver Cancer. 2012;1(3–4):183–9. Epub 2013/10/26. 10.1159/000343832 lic-0001-0183 [pii]. 24159583 PMC3760462

[19] Kaido T, Ogawa K, Mori A, Fujimoto Y, Ito T, Tomiyama K, et al Usefulness of the Kyoto criteria as expanded selection criteria for liver transplantation for hepatocellular carcinoma. Surgery. 2013;154(5):1053–60. Epub 2013/10/01. 10.1016/j.surg.2013.04.056 S0039-6060(13)00203-1 [pii]. . 24074704

[20] Yao FY, Ferrell L, Bass NM, Watson JJ, Bacchetti P, Venook A, et al Liver transplantation for hepatocellular carcinoma: expansion of the tumor size limits does not adversely impact survival. Hepatology. 2001;33(6):1394–403. Epub 2001/06/08. doi: S0270913901475495 [pii] 10.1053/jhep.2001.24563 . 11391528

[21] Roayaie S, Frischer JS, Emre SH, Fishbein TM, Sheiner PA, Sung M, et al Long-term results with multimodal adjuvant therapy and liver transplantation for the treatment of hepatocellular carcinomas larger than 5 centimeters. Ann Surg. 2002;235(4):533–9. Epub 2002/03/30. 11923610 10.1097/00000658-200204000-00012 PMC1422469

[22] Yao FY, Xiao L, Bass NM, Kerlan R, Ascher NL, Roberts JP. Liver transplantation for hepatocellular carcinoma: validation of the UCSF-expanded criteria based on preoperative imaging. American journal of transplantation: official journal of the American Society of Transplantation and the American Society of Transplant Surgeons. 2007;7(11):2587–96. 10.1111/j.1600-6143.2007.01965.x . 17868066

[23] Cillo U, Vitale A, Bassanello M, Boccagni P, Brolese A, Zanus G, et al Liver transplantation for the treatment of moderately or well-differentiated hepatocellular carcinoma. Ann Surg. 2004;239(2):150–9. Epub 2004/01/28. 10.1097/01.sla.0000109146.72827.76 14745321 PMC1356206

[24] Tamura S, Kato T, Berho M, Misiakos EP, O'Brien C, Reddy KR, et al Impact of histological grade of hepatocellular carcinoma on the outcome of liver transplantation. Arch Surg. 2001;136(1):25–30; discussion 1. Epub 2001/01/13. doi: soa0085 [pii]. . 11146770

[25] Xu X, Lu D, Ling Q, Wei X, Wu J, Zhou L, et al Liver transplantation for hepatocellular carcinoma beyond the Milan criteria. Gut. 2015 10.1136/gutjnl-2014-308513 . 25804634 PMC4893115

[26] Davison JM, Choudry HA, Pingpank JF, Ahrendt SA, Holtzman MP, Zureikat AH, et al Clinicopathologic and molecular analysis of disseminated appendiceal mucinous neoplasms: identification of factors predicting survival and proposed criteria for a three-tiered assessment of tumor grade. Mod Pathol. 2014 Epub 2014/03/19. 10.1038/modpathol.2014.37 modpathol201437 [pii]. . 24633196

[27] Zhou Z, Chen Z, Chen M, Wang R, Yin Y, Yao Y. Clinicopathologic factors predicting outcomes in patients with gastrointestinal stromal tumors of the rectum and colon. Tumour Biol. 2013 Epub 2014/01/01. 10.1007/s13277-013-1572-7 . 24375257

[28] Kenmochi K, Sugihara S, Kojiro M. Relationship of histologic grade of hepatocellular carcinoma (HCC) to tumor size, and demonstration of tumor cells of multiple different grades in single small HCC. Liver. 1987;7(1):18–26. Epub 1987/02/01. . 3033422 10.1111/j.1600-0676.1987.tb00310.x

[29] Barreto SG, Brooke-Smith M, Dolan P, Wilson TG, Padbury RT, Chen JW. Cirrhosis and microvascular invasion predict outcomes in hepatocellular carcinoma. ANZ J Surg. 2013;83(5):331–5. Epub 2012/09/05. 10.1111/j.1445-2197.2012.06196.x . 22943449

[30] Shah SA, Cleary SP, Wei AC, Yang I, Taylor BR, Hemming AW, et al Recurrence after liver resection for hepatocellular carcinoma: risk factors, treatment, and outcomes. Surgery. 2007;141(3):330–9. Epub 2007/03/14. doi: S0039-6060(06)00471-5 [pii] 10.1016/j.surg.2006.06.028 . 17349844

[31] Ikai I, Arii S, Kojiro M, Ichida T, Makuuchi M, Matsuyama Y, et al Reevaluation of prognostic factors for survival after liver resection in patients with hepatocellular carcinoma in a Japanese nationwide survey. Cancer. 2004;101(4):796–802. Epub 2004/08/12. 10.1002/cncr.20426 . 15305412

[32] Iwatsuki S, Dvorchik I, Marsh JW, Madariaga JR, Carr B, Fung JJ, et al Liver transplantation for hepatocellular carcinoma: a proposal of a prognostic scoring system. J Am Coll Surg. 2000;191(4):389–94. Epub 2000/10/13. doi: S1072-7515(00)00688-8 [pii]. 11030244 10.1016/s1072-7515(00)00688-8 PMC2966013

[33] Esnaola NF, Lauwers GY, Mirza NQ, Nagorney DM, Doherty D, Ikai I, et al Predictors of microvascular invasion in patients with hepatocellular carcinoma who are candidates for orthotopic liver transplantation. J Gastrointest Surg. 2002;6(2):224–32; discussion 32. Epub 2002/05/07. doi: S1091255X01000154 [pii]. . 11992808 10.1016/s1091-255x(01)00015-4

[34] Han DH, Choi GH, Kim KS, Choi JS, Park YN, Kim SU, et al Prognostic significance of the worst grade in hepatocellular carcinoma with heterogeneous histologic grades of differentiation. J Gastroenterol Hepatol. 2013;28(8):1384–90. Epub 2013/03/23. 10.1111/jgh.12200 . 23517197

[35] Perrault J, McGill DB, Ott BJ, Taylor WF. Liver biopsy: complications in 1000 inpatients and outpatients. Gastroenterology. 1978;74(1):103–6. Epub 1978/01/01. doi: S0016508578000074 [pii]. . 618417

[36] Froehlich F, Lamy O, Fried M, Gonvers JJ. Practice and complications of liver biopsy. Results of a nationwide survey in Switzerland. Dig Dis Sci. 1993;38(8):1480–4. Epub 1993/08/01. . 8344104 10.1007/BF01308607

[37] Durand F, Regimbeau JM, Belghiti J, Sauvanet A, Vilgrain V, Terris B, et al Assessment of the benefits and risks of percutaneous biopsy before surgical resection of hepatocellular carcinoma. J Hepatol. 2001;35(2):254–8. Epub 2001/10/03. doi: S0168-8278(01)00108-8 [pii]. . 11580148 10.1016/s0168-8278(01)00108-8

[38] Frilling A, Broelsch CE. Resection of hepatocellular carcinoma with out preoperative tumor biopsy. Ann Surg. 2002;235(4):604; author reply -5. Epub 2002/03/30. 11923620 10.1097/00000658-200204000-00023 PMC1422481

[39] Ng KK, Poon RT, Lo CM, Liu CL, Lam CM, Ng IO, et al Impact of preoperative fine-needle aspiration cytologic examination on clinical outcome in patients with hepatocellular carcinoma in a tertiary referral center. Arch Surg. 2004;139(2):193–200. Epub 2004/02/11. 10.1001/archsurg.139.2.193 139/2/193 [pii]. . 14769580

[40] Braune C, Widjaja A, Bartels M, Bleck JS, Flemming P, Manns MP, et al Surgical removal of a distinct subcutaneous metastasis of multilocular hepatocellular carcinoma 2 months after initial percutaneous ethanol injection therapy. Z Gastroenterol. 2001;39(9):789–92. Epub 2001/09/15. 10.1055/s-2001-17188 . 11558070

[41] Uenishi T, Kubo S, Hirohashi K, Tanaka H, Ohba K, Kinoshita H. Successful treatment of dissemination of hepatocellular carcinoma to the pleura and diaphragm after percutaneous liver biopsy. Dig Surg. 2001;18(3):225–7. Epub 2001/07/21. doi: 50137 [pii] 50137. . 11464017 10.1159/000050137

