# Supplementary figures and images for: Diagnostic Value of Preoperative Needle Biopsy for Tumor Grading Assessment in Hepatocellular Carcinoma
Source: PLoS One. 2015 Dec 14;10(12):e0144216. doi: 10.1371/journal.pone.0144216 (PMC4682812; doi:10.1371/journal.pone.0144216)

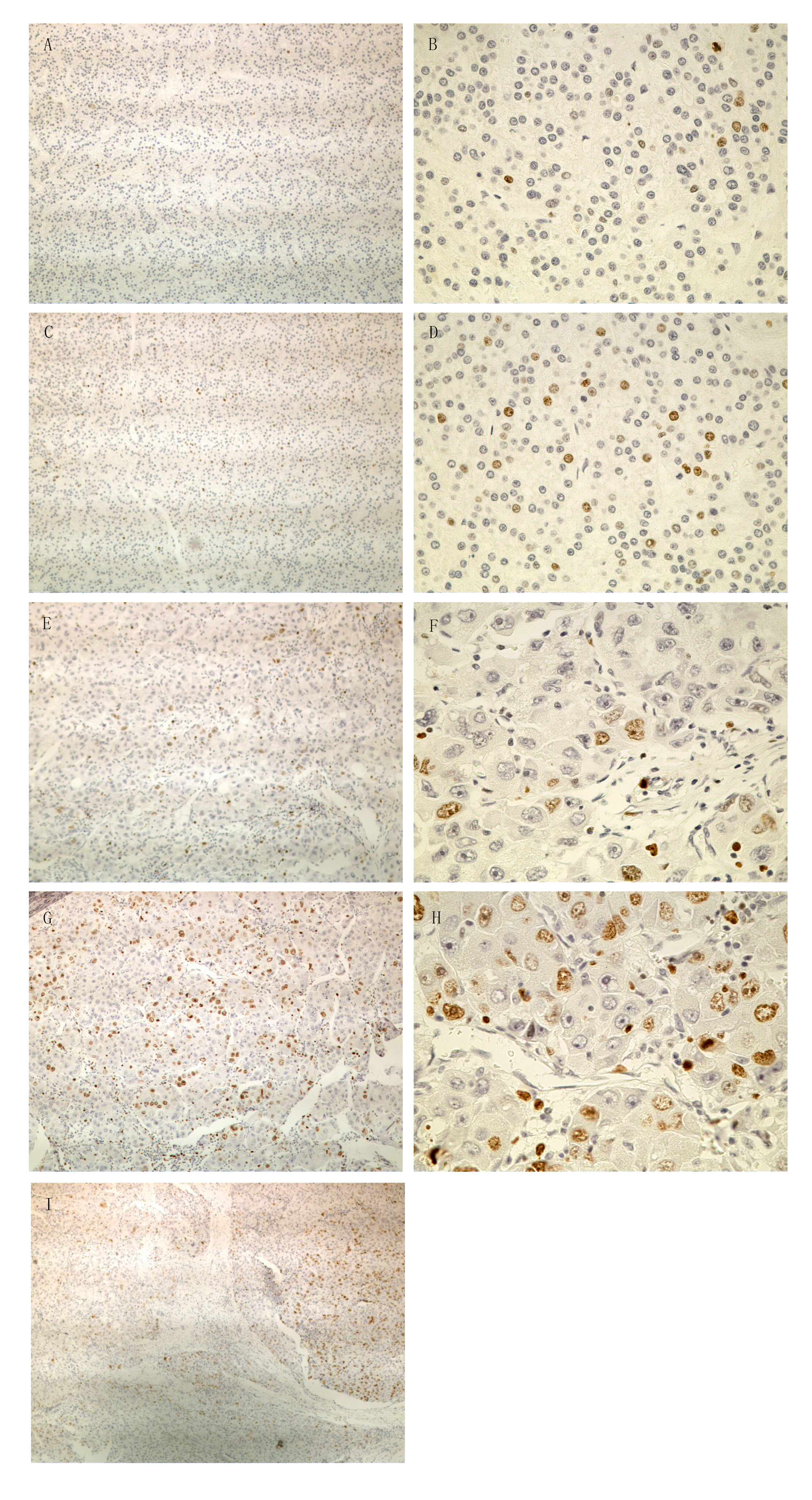

Supplement: S1 Fig — A and C: the histological grading of HCC cells were grade 2, the percentage of Ki-67 positive cell were <5% and 10%, respectively. E and G: The histological grading of HCC cells were grade 3 and the percentage of Ki-67 positive cell were 10% and 30%, respectively. I: histological grading of HCC was grade 3 in the overall lesion, whereas Ki-67 positive cell were uneven distributed. B, D, F and H demonstrated the higher magnification of A, C, E and G, respectively. (A, C, E, G and I: 200 ×magnification; B, D, F and H: with 400 × magnification). (TIF) [file pone.0144216.s001.tif]
